# Impact of amyloid and cardiometabolic risk factors on prognostic capacity of plasma neurofilament light chain for neurodegeneration

**DOI:** 10.1186/s13195-024-01564-y

**Published:** 2024-09-12

**Authors:** Keun You Kim, Eosu Kim, Jun-Young Lee, for the Alzheimer’s Disease Neuroimaging Initiative, Michael Weiner, Paul Aisen, Ronald Petersen, Clifford R. Jack, William Jagust, John Q. Trojanowki, Arthur W. Toga, Laurel Beckett, Robert C. Green, Andrew J. Saykin, John Morris, Leslie M. Shaw, Enchi Liu, Tom Montine, Ronald G. Thomas, Michael Donohue, Sarah Walter, Devon Gessert, Tamie Sather, Gus Jiminez, Danielle Harvey, Michael Donohue, Matthew Bernstein, Nick Fox, Paul Thompson, Norbert Schuff, Charles DeCArli, Bret Borowski, Jeff Gunter, Matt Senjem, Prashanthi Vemuri, David Jones, Kejal Kantarci, Chad Ward, Robert A. Koeppe, Norm Foster, Eric M. Reiman, Kewei Chen, Chet Mathis, Susan Landau, Nigel J. Cairns, Erin Householder, Lisa Taylor Reinwald, Virginia Lee, Magdalena Korecka, Michal Figurski, Karen Crawford, Scott Neu, Tatiana M. Foroud, Steven Potkin, Li Shen, Faber Kelley, Sungeun Kim, Kwangsik Nho, Zaven Kachaturian, Richard Frank, Peter J. Snyder, Susan Molchan, Jeffrey Kaye, Joseph Quinn, Betty Lind, Raina Carter, Sara Dolen, Lon S. Schneider, Sonia Pawluczyk, Mauricio Beccera, Liberty Teodoro, Bryan M. Spann, James Brewer, Helen Vanderswag, Adam Fleisher, Judith L. Heidebrink, Joanne L. Lord, Ronald Petersen, Sara S. Mason, Colleen S. Albers, David Knopman, Kris Johnson, Rachelle S. Doody, Javier Villanueva Meyer, Munir Chowdhury, Susan Rountree, Mimi Dang, Yaakov Stern, Lawrence S. Honig, Karen L. Bell, Beau Ances, John C. Morris, Maria Carroll, Sue Leon, Erin Householder, Mark A. Mintun, Stacy Schneider, Angela Oliver, Daniel Marson, Randall Griffith, David Clark, David Geldmacher, John Brockington, Erik Roberson, Hillel Grossman, Effie Mitsis, Leyla de Toledo-Morrell, Raj C. Shah, Ranjan Duara, Daniel Varon, Maria T. Greig, Peggy Roberts, Marilyn Albert, Chiadi Onyike, Daniel D’Agostino, Stephanie Kielb, James E. Galvin, Dana M. Pogorelec, Brittany Cerbone, Christina A. Michel, Henry Rusinek, Mony J. de Leon, Lidia Glodzik, Susan De Santi, P. Murali Doraiswamy, Jeffrey R. Petrella, Terence Z. Wong, Steven E. Arnold, Jason H. Karlawish, David Wolk, Charles D. Smith, Greg Jicha, Peter Hardy, Partha Sinha, Elizabeth Oates, Gary Conrad, Oscar L. Lopez, Mary Ann Oakley, Donna M. Simpson, Anton P. Porsteinsson, Bonnie S. Goldstein, Kim Martin, Kelly M. Makino, M. Saleem Ismail, Connie Brand, Ruth A. Mulnard, Gaby Thai, Catherine Mc Adams Ortiz, Kyle Womack, Dana Mathews, Mary Quiceno, Ramon Diaz Arrastia, Richard King, Myron Weiner, Kristen Martin Cook, Michael DeVous, Allan I. Levey, James J. Lah, Janet S. Cellar, Jeffrey M. Burns, Heather S. Anderson, Russell H. Swerdlow, Liana Apostolova, Kathleen Tingus, Ellen Woo, Daniel H. S. Silverman, Po H. Lu, George Bartzokis, Neill R. Graff Radford, Francine Parfitt, Tracy Kendall, Heather Johnson, Martin R. Farlow, Ann Marie Hake, Brandy R. Matthews, Scott Herring, Cynthia Hunt, Christopher H. van Dyck, Richard E. Carson, Martha G. MacAvoy, Howard Chertkow, Howard Bergman, Chris Hosein, Sandra Black, Bojana Stefanovic, Curtis Caldwell, Ging Yuek Robin Hsiung, Howard Feldman, Benita Mudge, Michele Assaly, Andrew Kertesz, John Rogers, Dick Trost, Charles Bernick, Donna Munic, Diana Kerwin, Marek Marsel Mesulam, Kristine Lipowski, Chuang Kuo Wu, Nancy Johnson, Carl Sadowsky, Walter Martinez, Teresa Villena, Raymond Scott Turner, Kathleen Johnson, Brigid Reynolds, Reisa A. Sperling, Keith A. Johnson, Gad Marshall, Meghan Frey, Jerome Yesavage, Joy L. Taylor, Barton Lane, Allyson Rosen, Jared Tinklenberg, Marwan N. Sabbagh, Christine M. Belden, Sandra A. Jacobson, Sherye A. Sirrel, Neil Kowall, Ronald Killiany, Andrew E. Budson, Alexander Norbash, Patricia Lynn Johnson, Thomas O. Obisesan, Saba Wolday, Joanne Allard, Alan Lerner, Paula Ogrocki, Leon Hudson, Evan Fletcher, Owen Carmichael, John Olichney, Charles DeCarli, Smita Kittur, Michael Borrie, T. Y. Lee, Rob Bartha, Sterling Johnson, Sanjay Asthana, Cynthia M. Carlsson, Steven G. Potkin, Adrian Preda, Dana Nguyen, Pierre Tariot, Adam Fleisher, Stephanie Reeder, Vernice Bates, Horacio Capote, Michelle Rainka, Douglas W. Scharre, Maria Kataki, Anahita Adeli, Earl A. Zimmerman, Dzintra Celmins, Alice D. Brown, Godfrey D. Pearlson, Karen Blank, Karen Anderson, Robert B. Santulli, Tamar J. Kitzmiller, Eben S. Schwartz, Kaycee M. Sink, Jeff D. Williamson, Pradeep Garg, Franklin Watkins, Brian R. Ott, Henry Querfurth, Geoffrey Tremont, Stephen Salloway, Paul Malloy, Stephen Correia, Howard J. Rosen, Bruce L. Miller, Jacobo Mintzer, Kenneth Spicer, David Bachman, Elizabether Finger, Stephen Pasternak, Irina Rachinsky, John Rogers, Andrew Kertesz, Dick Drost, Nunzio Pomara, Raymundo Hernando, Antero Sarrael, Susan K. Schultz, Laura L. Boles Ponto, Hyungsub Shim, Karen Elizabeth Smith, Norman Relkin, Gloria Chaing, Lisa Raudin, Amanda Smith, Kristin Fargher, Balebail Ashok Raj

**Affiliations:** 1 Department of Psychiatry, Seoul Metropolitan Government - Seoul National University (SMG-SNU) Boramae Medical Center, Seoul National University College of Medicine, 20 Boramae-Ro 5-Gil, Dongjak-Gu, Seoul, 07061 Republic of Korea; 2 Department of Psychiatry, Institute of Behavioral Science in Medicine, Yonsei University College of Medicine, 50-1 Yonsei-Ro, Seodaemun-Gu, Seoul, 03722 Republic of Korea; 3 Brain Korea 21 FOUR Project for Medical Science, Yonsei University College of Medicine, 50-1 Yonsei-Ro, Seodaemun-Gu, Seoul, 03722 Republic of Korea

**Keywords:** Neurofilament light chain, Alzheimer’s disease, Blood-based biomarker, Dementia, Prognosis, Cardiovascular disease, Metabolic syndrome, Kidney disease

## Abstract

**Background:**

Plasma neurofilament light chain (NfL) is a blood biomarker of neurodegeneration, including Alzheimer’s disease. However, its usefulness may be influenced by common conditions in older adults, including amyloid-β (Aβ) deposition and cardiometabolic risk factors like hypertension, diabetes mellitus (DM), impaired kidney function, and obesity. This longitudinal observational study using the Alzheimer’s Disease Neuroimaging Initiative cohort investigated how these conditions influence the prognostic capacity of plasma NfL.

**Methods:**

Non-demented participants (cognitively unimpaired or mild cognitive impairment) underwent repeated assessments including the Alzheimer’s Disease Assessment Scale-Cognitive subscale (ADAS-Cog) scores, hippocampal volumes, and white matter hyperintensity (WMH) volumes at 6- or 12-month intervals. Linear mixed-effect models were employed to examine the interaction between plasma NfL and various variables of interest, such as Aβ (evaluated using Florbetapir positron emission tomography), hypertension, DM, impaired kidney function, or obesity.

**Results:**

Over a mean follow-up period of 62.5 months, participants with a mean age of 72.1 years (*n* = 720, 48.8% female) at baseline were observed. Higher plasma NfL levels at baseline were associated with steeper increases in ADAS-Cog scores and WMH volumes, and steeper decreases in hippocampal volumes over time (all *p*-values < 0.001). Notably, Aβ at baseline significantly enhanced the association between plasma NfL and longitudinal changes in ADAS-Cog scores (*p*-value 0.005) and hippocampal volumes (*p*-value 0.004). Regarding ADAS-Cog score and WMH volume, the impact of Aβ was more prominent in cognitively unimpaired than in mild cognitive impairment. Hypertension significantly heightened the association between plasma NfL and longitudinal changes in ADAS-Cog scores, hippocampal volumes, and WMH volumes (all *p*-values < 0.001). DM influenced the association between plasma NfL and changes in ADAS-Cog scores (*p*-value < 0.001) without affecting hippocampal and WMH volumes. Impaired kidney function did not significantly alter the association between plasma NfL and longitudinal changes in any outcome variables. Obesity heightened the association between plasma NfL and changes in hippocampal volumes only (*p*-value 0.026).

**Conclusion:**

This study suggests that the prognostic capacity of plasma NfL may be amplified in individuals with Aβ or hypertension. This finding emphasizes the importance of considering these factors in the NfL-based prognostic model for neurodegeneration in non-demented older adults.

**Supplementary Information:**

The online version contains supplementary material available at 10.1186/s13195-024-01564-y.

## Background

Predicting central neurodegeneration at the preclinical stage is crucial for the prevention and early intervention of Alzheimer’s disease (AD), especially in the era of emerging disease-modifying treatments [1]. Neurofilament light chain (NfL), a subunit of neurofilaments abundant in neuronal axons, is a non-invasive blood-based biomarker for detecting or predicting neurodegeneration and clinical progression in preclinical or prodromal stage of dementia [2–10]. The Alzheimer’s Association Workgroup has recently updated the diagnostic and staging criteria for AD, including plasma NfL as one of the key blood-based biomarkers [11]. Classified as an “N (neurodegeneration)” biomarker, plasma NfL is useful for assessing the stage or prognosis of AD [11]. Furthermore, plasma NfL is highlighted as a cost-effective and non-invasive surrogate biomarker for clinical trials targeting the preclinical stage of dementia [12].

However, caution is required when interpreting the meaning of plasma NfL levels, as they can be influenced by various conditions commonly observed in older adults. Cerebral amyloid-β (Aβ) deposition, found in over one-third of cognitively unimpaired older adults [13], can accelerate the release of NfL into the bloodstream owing to its neurotoxicity [14]. Additionally, cardiometabolic risk factors, such as hypertension, diabetes mellitus (DM), impaired kidney function, and obesity, can influence NfL levels in the blood [2]. Hypertension-related cardiovascular disease and DM are associated with increased plasma NfL levels, which may be attributed to microvascular brain injury [15–17]. Cerebral small vessel disease, closely related to hypertension and DM, is also associated with increased plasma NfL levels [2, 18]. Moreover, previous studies have indicated that impaired kidney function was associated with elevated plasma NfL levels due to reduced clearance or metabolism of plasma NfL [15, 19]. Individuals with obesity or high body mass index (BMI) exhibit low blood NfL levels, which is possibly explained by the dilution of plasma NfL due to increased blood volume [15, 20].

Although these common old age-related conditions (Aβ and cardiometabolic risk factors) could confound the level of plasma NfL, their impact on the capacity of plasma NfL for predicting neurodegeneration and clinical progression remains unexplored. Previous longitudinal studies evaluating the association between baseline plasma NfL and neurodegenerative outcome did not consider the influence of Aβ and cardiometabolic risk factors [3–9]. It is important to find out which of these factors should be considered when establishing a model for predicting cognitive decline using plasma NfL. Using data from non-demented individuals, we investigated whether plasma NfL is differently associated with cognitive decline over time, depending on the statuses of Aβ and cardiometabolic conditions (hypertension, DM, impaired kidney function, or obesity). We also assessed changes in neuroimaging abnormalities by structural brain magnetic resonance imaging (MRI) to elucidate the underlying mechanism of cognitive decline.

## Methods

### Study participants

The data for this study were sourced from the Alzheimer’s Disease Neuroimaging Initiative (ADNI) database (http://adni.loni.usc.edu). ADNI is a longitudinal study that defines AD’s progression using biomarkers such as neuroimages (www.adni-info.org). Detailed inclusion and exclusion criteria for the study participants have been outlined elsewhere (https://adni.loni.usc.edu/methods/documents/) [21]. Individuals aged 55–90 years who met the following criteria were recruited: (i) minimal depression (score under 6 on the Short form of Geriatric Depression Scale [SGDS]); (ii) low vascular dementia risk (Hachinski Ischemic Score of 4 or below); (iii) stable permitted medications for 4 weeks, excluding psychoactive medications affecting cognitive function; (iv) no significant visual or auditory impairment that could interfere with neuropsychological tests; (v) availability of a study partner with at least 10 h/week of contact who could accompany to visit; (vi) at least 6 grades of education or work history; and (vii) fluency in English or Spanish. Exclusion criteria included: (i) significant neurologic diseases other than suspected AD (Parkinson’s disease, multi-infarct dementia, Huntington’s disease, normal pressure hydrocephalus, brain tumor, seizure disorder, hemorrhage, or known structural brain abnormalities); (ii) baseline MRI scan with evidence of infection, infarction, or other focal lesions; (iii) presence of pacemakers, aneurysm clips, artificial heart valves, ear implant, metal fragments, or other foreign objects in the body; (iv) history of major depression or bipolar disorder within a past year; (v) history of schizophrenia; (vi) history of alcohol or substance abuse or dependence within the past 2 years; and (vii) clinically significant abnormalities in vitamin B12 or thyroid function test.

**Fig. 1:**
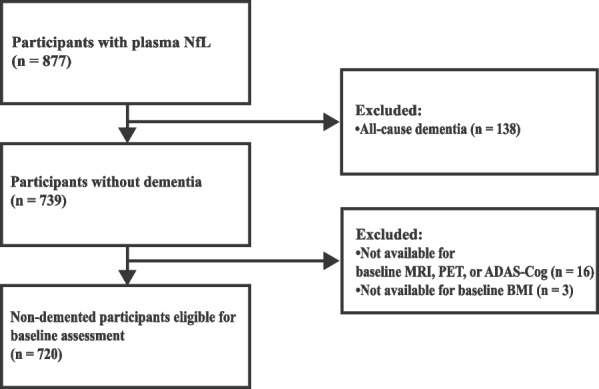
Selection of the study population. Abbreviations: ADAS-Cog, Alzheimer’s Disease Assessment Scale-Cognitive subscale; MRI, magnetic resonance imaging; NfL, neurofilament light chain; PET, positron emission tomography

Plasma NfL levels at baseline visits were measured between June 2010 and March 2022. Among the 877 participants who had their baseline plasma NfL level measured, 739 were free from dementia (cognitively unimpaired [CU] or mild cognitive impairment [MCI]). Criteria for dementia were previously described [21], based on the National Institute of Neurological and Communicative Disorders and Stroke–Alzheimer’s Disease and Related Disorders Association criteria for probable AD [22]. MCI participants met all the following criteria [21]: (i) subjective memory concern reported by subject, study partner, or clinician; (ii) Mini-Mental State Examination score between 24 and 30; (iii) the Clinical Dementia Rating score of 0.5 with a memory box score of 0.5 or higher; and (iv) objective memory impairment observed by education-adjusted scores on delayed recall of one paragraph from the Wechsler Memory Scale-Revised Logical Memory II subscale. Participants classified as CU had a Mini-Mental State Examination score between 24 to 30, a Clinical Dementia Rating score of 0, and objective normal memory function assessed by the delayed recall of one paragraph from the Wechsler Memory Scale-Revised Logical Memory II subscale. Among these non-demented participants (MCI or CU), we excluded those with missing data from Florbetapir positron emission tomography (PET), brain MRI, or cognitive tests (*n* = 16). After excluding three participants without data on baseline body mass index (BMI), final data from 720 participants were investigated (Fig. 1). The study was approved by the Institutional Review Board of each participating institution, and written informed consent was obtained from all participants.

### Cognitive function assessment

We used the Alzheimer’s Disease Assessment Scale-Cognitive (ADAS-Cog) to assess the cognitive function of each participant [23]. The ADAS-Cog comprises 13 tasks: word recall task, commands, constructional praxis, delayed word recall, naming task, ideational praxis, orientation, word recognition task, remembering test instructions, comprehension, word-finding difficulty, spoken language ability, and number cancellation. The ADAS-Cog provided a score of 0–85, where a higher score indicated a more prominent cognitive impairment. Each participant underwent the ADAS-Cog assessment every 6 or 12 months.

### Structural MRI procedure and analysis

All participants underwent 3.0 T brain MRI scans at each site using the ADNI GO/2 protocol (https://adni.loni.usc.edu/methods/mri-tool/mri-analysis/). The ADNI MRI Quality Control team at Mayo Clinic reviewed each scan. We tracked the changes in hippocampal and white matter hyperintensity (WMH) volumes, both of which are reported to be predicted by plasma NfL [7, 18]. Hippocampus is a commonly used region of interest for assessing AD-related neurodegeneration [3, 7, 14]. WMH is an indicator of cerebral small vessel disease, which is the most common coexisting pathology with AD and exacerbates cognitive decline [24, 25]. It also causes secondary grey and white matter loss in both directly and indirectly connected brain regions via compromised blood–brain barrier, impaired cerebral blood flow, and perivascular injury, resulting in neurodegeneration [24]. Hippocampus and WMH volumetric data were calculated by the University of California team at Davis using 3D T1 and T2 fluid-attenuated inversion recovery sequence images and the FSL toolbox. Similar to the ADAS-Cog, the MRI scans were repeated at 6 or 12 months.

### Baseline status of Aβ and cardiometabolic risk factors

Cortical Aβ analysis was based on the data from Florbetapir (18F-AV-45) PET conducted by the team at the University of California Berkeley processed with FreeSurfer v7.1.1 (https://surfer.nmr.mgh.harvard.edu/) [26]. The volume-weighted standard uptake value ratio (SUVR) of each cortical region was calculated after skull-stripping, segmentation, and delineating cortical and subcortical regions. The mean SUVR value of the frontal, lateral parietal, lateral temporal, and anterior/posterior cingulate regions relative to the whole cerebellum was regarded as the composite SUVR of each participant. According to the previous study [26], a composite SUVR ≥ 1.11 was considered as a cerebral Aβ ( +) status.

Participants were considered to have hypertension if they had a history of hypertension, a systolic blood pressure of 140 mmHg or higher and/or a diastolic blood pressure of 90 mmHg or higher, or if they were taking anti-hypertensive medication. This selection method was aligned with a widely used definition of hypertension in epidemiology [27]. Participants using anti-diabetic medications or having a fasting glucose level exceeding 126 mg/dL were categorized as having DM. Participants with a BMI of 30 kg/m^2^ or higher were classified as being obese. The presence of impaired kidney function was determined as one of the following: (i) a history of kidney disease (e.g., nephrectomy, nephritis, renal failure, or horseshoe kidney), or (ii) an estimated glomerular filtration rate (eGFR) under 60 mL/min/1.73 m^2^ calculated using the Chronic Kidney Disease Epidemiology Collaboration equation [28].

### Assessment of other covariates

Factors potentially affecting cognitive function or dementia progression were selected as covariates. The education level of each participant, which is closely associated with cognitive decline [29], was assessed by total years of education. The number of apolipoprotein E (APOE) ε4 alleles of each participant was used as a covariate, due to its relation to the increased risk of dementia [30]. Details on APOE genotyping are described at https://adni.loni.usc.edu/data-samples/data-types/genetic-data/. History of smoking and alcohol abuse, factors related to cognitive decline [31], was evaluated by self-reported records. Since depression is also a risk factor for dementia [30, 31], its severity was assessed using SGDS. Clinical cognitive status at baseline, such as CU or MCI, was included as a covariate, given its influence on the rate of cognitive decline or dementia progression [32].

### Blood sampling procedure and plasma NfL level measurement

Details of the blood sampling procedure and plasma NfL assay are described at www.adni-info.org. Blood samples were collected in EDTA tubes after overnight fasting for ≥ 6 h. After gently mixed by inversion 10–12 times, tubes were centrifuged at 3000 rpm for 15 min. Plasma was then transferred to a separate tube, immediately frozen by dry ice in each site, and housed in a -80 ℃ freezer until analysis. Plasma NfL levels were quantified at the Clinical Neurochemistry Laboratory at the University of Gothenburg, Sweden, using the Single Molecule array (Simoa) technique (Quanterix, Lexington, Massachusetts, United States) [14, 33]. The combination of monoclonal antibodies with bovine NfL as a calibrator was used, with an analytic sensitivity of < 1.0 pg/mL, and no sample exhibited plasma NfL levels below the limit of detection.

### Statistical analysis

The cross-sectional association of plasma NfL level and Aβ and cardiometabolic risk factors (hypertension, DM, impaired kidney function, and obesity) at baseline was assessed using a multiple linear regression model. The outcome variable was plasma NfL level, and the main explanatory variables were Florbetapir PET SUVR, systolic blood pressure, fasting glucose level, eGFR, and BMI. The model was adjusted for age, sex, years of education, APOE ε4 allele count, smoking history, alcohol abuse, SGDS, ADAS-Cog score, hippocampal volume, WMH volume, and clinical diagnosis of baseline cognitive status (CU or MCI). Missing data were handled by listwise deletion.

Subsequently, we evaluated the predictive value of plasma NfL for changes in cognition and brain structure (hippocampal and WMH volume) using linear mixed-effect models. Outcome variables included ADAS-Cog score, hippocampal volume, and WMH volume, with the main explanatory variable being the interaction term ‘plasma NfL level × time since baseline (months)’. We considered covariates such as age, sex, years of education, APOE ε4 allele count, smoking history, alcohol abuse, SGDS, Aβ status, hypertension, DM, impaired kidney function, obesity, and clinical diagnosis of baseline cognitive status (CU or MCI).

Additional linear mixed-effect models were applied to examine the impact of Aβ and cardiometabolic risk factors on the prognostic capacity of plasma NfL for changes in cognition and brain structure. Outcome variables were ADAS-Cog score, hippocampal volume, and WMH volume. Fixed effects included plasma NfL level, time since baseline, and the variable of interest (Aβ, hypertension, DM, impaired kidney function, and obesity), along with relevant interaction terms such as ‘plasma NfL × time × Aβ/hypertension/DM/impaired kidney function/obesity’. Covariates encompassed age, sex, years of education, APOE ε4 allele count, smoking history, alcohol abuse, SGDS, and clinical diagnosis of baseline cognitive status (CU or MCI). For Aβ, the same analyses were performed separately within MCI and CU participants to minimize the confounding effect of baseline clinical cognitive status. To examine the impact of each risk factor’s severity, additional sensitivity analyses were performed using the following continuous variables: systolic blood pressure, fasting glucose level, eGFR, and BMI. Since hypotension, hypoglycemia, glomerular hyperfiltration, and BMI loss can also potentially exacerbate cognitive decline [34–38], the impact of each continuous variable was analyzed within participants with the presence of a corresponding risk factor.

Among the three outcome variables, ADAS-Cog scores and WMH volumes underwent square root transformation due to their non-normal distribution. All continuous variables, except for time since baseline, were standardized to z-scores prior to analyses using the baseline mean and standard deviation of each variable. Statistical analyses were performed using R, version 4.3.1 (R Foundation for Statistical Computing), with a significance threshold set at a two-sided *p*-value of 0.05. The lme4 package, version 1.1–33, was used to fit linear mixed-effect models.

## Results

### Baseline characteristics of ADNI participants

Table 1 displays the baseline characteristics of the 720 study participants. The mean age was 72.1 years, with 351 (48.8%) being female. Among the non-demented participants, 441 (61.3%) were diagnosed with MCI. Aβ ( +) was observed in 341 (47.4%) participants, while 478 (66.4%) had hypertension, 114 (15.8%) had DM, 39 (5.4%) had impaired kidney function, and 186 (25.8%) had obesity. Supplementary Table 1 provides the number of participants who underwent ADAS-Cog and MRI at specific time points.

**Table 1:** Demographic and clinical characteristics of non-demented participants at baseline

	Overall participants (*n* = 720)
Age (years)	72.1 (7.00)
Sex, female	351 (48.8%)
Education (years)	16.4 (2.60)
Race/ethnicity, non-Hispanic White	663 (92.1%)
Cognitive status	
CU	279 (38.8%)
MCI	441 (61.3%)
APOE ε4 allele count	
0	425 (59.0%)
1	243 (33.8%)
2	52 (7.2%)
History of ever smoking	136 (18.9%)
History of alcohol abuse	17 (2.4%)
SGDS	1.40 (1.42)
Follow-up period (months)	62.5 (35.9)
Florbetapir PET SUVR	1.18 (0.216)
Cerebral Aβ status ( +)^a^	341 (47.4%)
Hypertension	478 (66.4%)
Well-controlled hypertension^b^	104 (21.8% of hypertension)
DM	114 (15.8%)
Impaired kidney function	39 (5.4%)
Obesity^c^	186 (25.8%)
ADAS-Cog score	12.5 (6.60)
Hippocampal volume (mm^3^)	6490 (854)
WMH volume (mm^3^)	6520 (9430)
Plasma NfL (pg/mL)	36.8 (20.3)

Data are presented as mean (standard deviation) for continuous variables and n (%) for categorical variables

*Abbreviations*: *Aβ* amyloid-β, *ADAS-Cog* Alzheimer's Disease Assessment Scale-Cognitive subscale, *APOE* apolipoprotein E, *BMI* body mass index, *CU* cognitively unimpaired, *DM* diabetes mellitus, *MCI* mild cognitive impairment, *NfL* neurofilament light chain, *PET* positron emission tomography, *SGDS* Short form of Geriatric Depression Scale, *SUVR* standard uptake value ratio, *WMH* white matter hyperintensity

^a^Florbetapir PET SUVR 1.11 or over was regarded as cerebral Aβ status ( +)

^b^Systolic blood pressure under 120 mmHg, in combination with history of hypertension or concurrent anti-hypertensive medication, was defined as well-controlled hypertension

^c^BMI 30 kg/m^2^ or over was defined as obesity

### Cross-sectional associations of plasma NfL with Aβ and cardiometabolic risk factors at baseline

Supplementary Table 2 presents the result of a multiple regression model, where the outcome variable was the plasma NfL level. After adjusting for covariates, decreased eGFR (beta -0.236, *p*-value < 0.001) and decreased BMI (beta -0.152, *p*-value < 0.001) were significantly associated with increased plasma NfL levels, respectively. SUVR, systolic blood pressure, and fasting glucose level were not significantly associated with plasma NfL levels (*p*-values > 0.05).

### Associations between plasma NfL and longitudinal changes in ADAS-Cog scores, hippocampal volumes, and WMH volumes

**Fig. 2:**
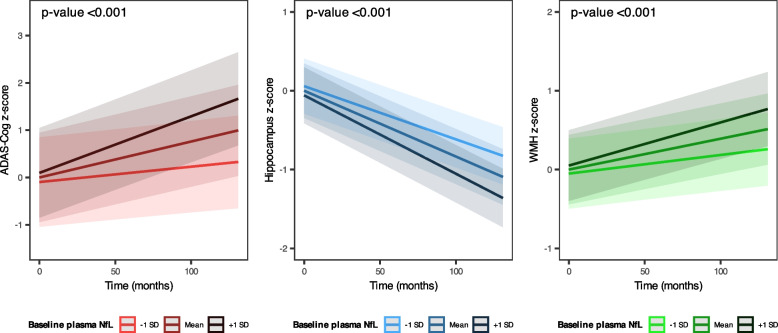
Associations between baseline NfL levels and longitudinal changes in ADAS-Cog scores, hippocampal volumes, and WMH volumes. Data show the associations between baseline plasma NfL and longitudinal changes in ADAS-Cog scores (left panel), hippocampal volumes (middle panel), and WMH volumes (right panel). Higher baseline plasma NfL levels were associated with steeper increases in ADAS-cog scores and WMH volumes, and steeper decreases in hippocampal volumes over time (all *p*-values < 0.001). Of outcome variables, ADAS-Cog score and WMH volume were square root transformed due to non-normal distribution. Continuous variables, including plasma NfL level and outcome variables, were standardized to z-scores. The plotted lines represent estimated z-scores of ADAS-Cog scores, hippocampal volumes, or WMH volumes over time under the condition of baseline plasma NfL at mean -1SD, mean, and mean + 1SD. *P*-values were calculated to identify the significance of the two-way interaction term including baseline NfL level and time. Models were adjusted for the following covariates: baseline age, sex, years of education, APOE ε4 allele count, ever smoking, alcohol abuse, SGDS, Aβ status, hypertension, DM, impaired kidney function, obesity, and baseline cognitive status (MCI or CU). Abbreviations: ADAS-Cog, Alzheimer’s Disease Assessment Scale-Cognitive subscale; APOE, apolipoprotein E; CU, cognitively unimpaired; DM, diabetes mellitus; MCI, mild cognitive impairment; NfL, neurofilament light chain; SD, standard deviation; SGDS, Short form of Geriatric Depression Scale; SUVR, standard uptake value ratio; WMH, white matter hyperintensity

After adjusting for covariates, plasma NfL levels were significantly associated with longitudinal changes in ADAS-Cog scores, hippocampal volumes, and WMH volumes (all *p*-values < 0.001, Supplementary Table 3). In detail, higher plasma NfL levels were significantly associated with faster increases in ADAS-Cog scores (left panel) and WMH volumes (right panel), and faster decreases in hippocampal volumes (middle panel, Fig. 2).

### Impact of Aβ and cardiometabolic risk factors on associations between plasma NfL and longitudinal changes in ADAS-Cog scores, hippocampal volumes, and WMH volumes

#### Aβ

**Fig. 3:**
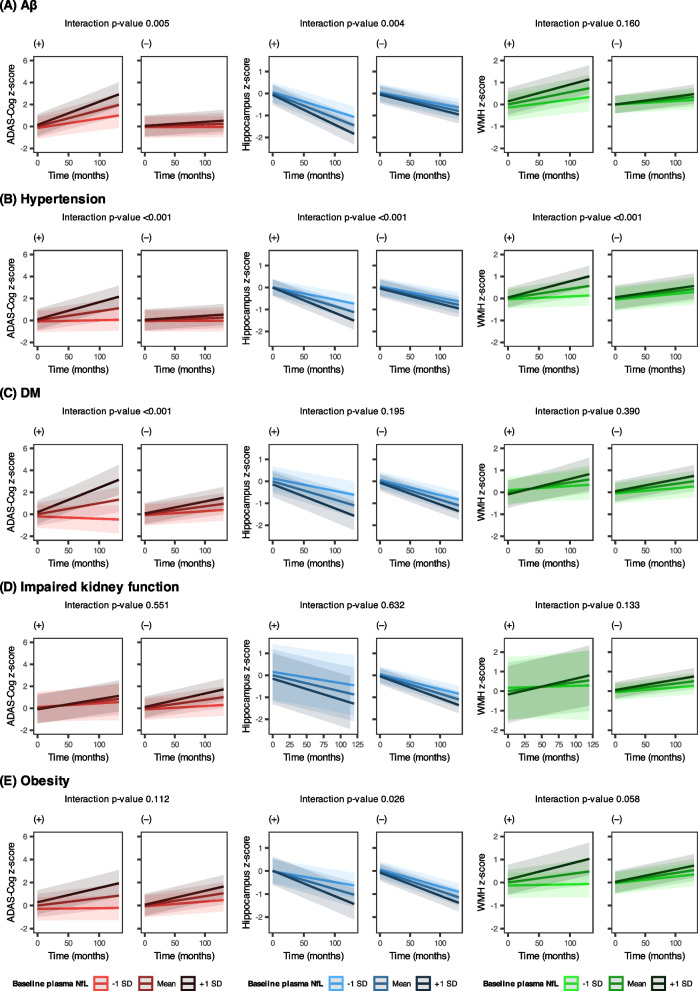
Associations between baseline plasma NfL and longitudinal changes in ADAS-Cog scores, hippocampal volumes, or WMH volumes: stratified by the status of Aβ and cardiometabolic risk factors. Data show how the associations between plasma NfL and longitudinal changes in ADAS-Cog scores (left panel), hippocampal volumes (middle panel), and WMH volumes (right panel) were affected by the Aβ or cardiometabolic risk factors. **A** Aβ significantly moderated the association between plasma NfL and longitudinal ADAS-Cog scores (*p*-value 0.005) and hippocampal volumes (*p*-value 0.004), not WMH volumes (*p*-value 0.160). Specifically, while higher baseline plasma NfL levels were associated with faster increases in ADAS-Cog scores and decreases in hippocampal volumes, the magnitude of these changes in slopes was more pronounced in Aβ ( +) status compared to Aβ ( −) status. **B** Similarly, hypertension significantly moderated the association between plasma NfL and longitudinal changes in all outcome variables (all *p*-values < 0.001). **C** DM significantly affected the association between plasma NfL and longitudinal ADAS-Cog scores (*p*-value < 0.001) without affecting hippocampal and WMH volumes. **D** Impaired kidney function did not affect the association between plasma NfL and any outcome variables (all *p*-values > 0.05). **E** Obesity significantly moderated the association between plasma NfL and longitudinal hippocampal volumes (*p*-value 0.026) without affecting ADAS-Cog scores (*p*-value 0.112) and WMH volumes (*p*-value 0.058). Of outcome variables, ADAS-Cog score and WMH volume were square root transformed due to non-normal distribution. Continuous variables, including plasma NfL level and outcome variables, were standardized to z-scores. The plotted lines represent estimated z-scores of ADAS-Cog scores, hippocampal volumes, or WMH volumes over time under the condition of baseline plasma NfL at mean -1SD, mean, and mean + 1SD. Interaction *p*-values were calculated to identify the significance of the three-way interaction term including baseline NfL, time, and the variable of interest (Aβ, hypertension, DM, impaired kidney function, or obesity). Abbreviations: Aβ, amyloid-β; ADAS-Cog, Alzheimer’s Disease Assessment Scale-Cognitive subscale; DM, diabetes mellitus; NfL, neurofilament light chain; SD, standard deviation; WMH, white matter hyperintensity

The interaction term ‘plasma NfL × time × Aβ’ revealed significant associations with ADAS-Cog scores and hippocampal volumes, but not with WMH volumes (Table 2, see Supplementary Table 4 for detailed parameter estimates). These results imply that the associations between baseline plasma NfL and the changes in ADAS-Cog scores, hippocampal volume, and WMH volumes were influenced by Aβ status. Specifically, compared to Aβ (–) participants, Aβ ( +) participants demonstrated more pronounced changes in the slopes of ADAS-Cog score and hippocampal volume as the plasma NfL level increased (Fig. 3A, left and middle panel). For example, the slope in ADAS-Cog z-score changed from 0.0087/month in participants with low plasma NfL (mean – 1SD) to 0.0213/month in those with high plasma NfL (mean + 1 SD) among participants with Aβ ( +). This change was greater than that in Aβ (–) participants (changing from 0.0002/month to 0.0036/month, Supplementary Table 5). Supplementary Table 5 also presents the estimated rates of change in hippocampal and WMH volume, stratified by baseline plasma NfL level and Aβ status.

**Table 2:** Impact of Aβ and cardiometabolic risk factors on associations between baseline plasma NfL and longitudinal changes in ADAS-Cog scores, hippocampal volumes, or WMH volumes

Explanatory variable	Outcome	beta	t value	*p*-value
Plasma NfL × Time × Aβ	ADAS-Cog score	0.004	2.797	0.005
	Hippocampal volume	-0.002	-2.922	0.004
	WMH volume	0.001	1.408	0.160
Plasma NfL × Time × Hypertension	ADAS-Cog score	0.005	3.606	< 0.001
	Hippocampal volume	-0.002	-3.814	< 0.001
	WMH volume	0.002	3.389	< 0.001
Plasma NfL × Time × DM	ADAS-Cog score	0.008	3.436	< 0.001
	Hippocampal volume	-0.001	-1.296	0.195
	WMH volume	0.001	0.861	0.390
Plasma NfL × Time × Impaired kidney function	ADAS-Cog score	-0.002	-0.597	0.551
	Hippocampal volume	-0.001	-0.480	0.632
	WMH volume	0.002	1.502	0.133
Plasma NfL × Time × Obesity	ADAS-Cog score	0.003	1.594	0.112
	Hippocampal volume	-0.002	-2.238	0.026
	WMH volume	0.002	1.898	0.058

Shown are results of linear mixed-effect models where each main explanatory variable was the three-way interaction term including baseline NfL, time, and the variable of interest (Aβ, hypertension, DM, impaired kidney function, or obesity). If the interaction term is statistically significant (*p*-value < 0.05), the association between plasma NfL and longitudinal changes in outcome is dependent on the status of the variable of interest (Aβ, hypertension, DM, impaired kidney function, or obesity)

Of outcome variables, ADAS-Cog score and WMH volume were square root transformed due to non-normal distribution

Continuous variables except for time were standardized to z-scores

All models were adjusted for the following covariates: baseline age, sex, years of education, APOE ε4 allele count, ever smoking, alcohol abuse, SGDS, Aβ status, hypertension, DM, obesity, impaired kidney function, and baseline cognitive status (MCI or CU)

Florbetapir PET SUVR 1.11 or over was regarded as Aβ ( +) status

*Abbreviations*: *Aβ* amyloid-β, *ADAS-Cog* Alzheimer’s Disease Assessment Scale-Cognitive subscale, *APOE* apolipoprotein E, *CU* cognitively unimpaired, *DM* diabetes mellitus, *MCI* mild cognitive impairment, *NfL* neurofilament light chain, *PET* positron emission tomography, *SGDS* Short form of Geriatric Depression Scale, *SUVR* standard uptake value ratio, *WMH* white matter hyperintensity

##### Subgroup analysis: impact of Aβ in CU or in MCI

Table 3 depicts the results of subgroup analyses stratified by baseline cognitive status (CU or MCI). In MCI participants (*n* = 441), Aβ did not affect the association between baseline plasma NfL and longitudinal changes in any outcome variables (all *p*-values > 0.05). However, in CU participants (*n* = 279), Aβ significantly moderated the association between plasma NfL and longitudinal ADAS-Cog scores (beta 0.005, *p*-value 0.007) and WMH volumes (beta 0.003, *p*-value 0.036).

**Table 3:** Impact of Aβ on association between baseline plasma NfL and longitudinal cognition/brain structure, stratified by baseline cognitive status (MCI vs CU)

Explanatory variable	Outcome	beta	t value	*p*-value
Plasma NfL × Time × Aβ				
In MCI participants (*n* = 441)	ADAS-Cog score	0.0004	0.173	0.863
	Hippocampal volume	-0.001	-0.954	0.341
	WMH volume	-0.001	-1.009	0.314
In CU participants (*n* = 279)	ADAS-Cog score	0.005	2.724	0.007
	Hippocampal volume	-0.002	-1.519	0.130
	WMH volume	0.003	2.117	0.036

Shown are results of linear mixed-effect models where the main explanatory variable was the three-way interaction term, including baseline plasma NfL, time, and Aβ, analyzed using the entire dataset and stratified by baseline cognitive status (MCI or CU). If the interaction term is statistically significant (*p*-value < 0.05), the association between plasma NfL and longitudinal changes in outcome is dependent on the status of Aβ

Of outcome variables, ADAS-Cog score and WMH volume were square root transformed due to non-normal distribution

All continuous variables were standardized to z-scores for comparison between models

The model using entire participants was adjusted for the following covariates: baseline age, sex, years of education, APOE ε4 allele count, ever smoking, alcohol abuse, SGDS, Aβ status, hypertension, DM, obesity, impaired kidney function, and baseline cognitive status (MCI or CU). Models stratified by MCI or CU were adjusted for same covariates except for baseline cognitive status

*Abbreviations*: *Aβ* amyloid-β, *ADAS-Cog* Alzheimer’s Disease Assessment Scale-Cognitive subscale, *APOE* apolipoprotein E, *CU* cognitively unimpaired, *DM* diabetes mellitus, *MCI* mild cognitive impairment, *NfL* neurofilament light chain, *PET* positron emission tomography, *SGDS* Short form of Geriatric Depression Scale, *SUVR* standard uptake value ratio, *WMH* white matter hyperintensity

#### Cardiometabolic risk factors (Hypertension, DM, impaired kidney function, and obesity)

Similar to Aβ, hypertension status altered the longitudinal association between baseline plasma NfL and ADAS-Cog score, hippocampal volume, and WMH volume (Table 2, see Supplementary Table 6 for detailed parameter estimates). Figure 3B illustrates this trend; the magnitudes of changes in slopes of ADAS-Cog scores, hippocampal volumes, and WMH volumes alongside increasing plasma NfL levels were more marked in hypertension group compared to non-hypertension group. Supplementary Table 7 depicts the estimated slopes and standard errors in ADAS-Cog score, hippocampal volume, and WMH volume at different baseline plasma NfL levels (mean – 1SD, mean, and mean + 1SD) stratified by hypertension status.

Unlike Aβ and hypertension, DM exclusively influenced the association between baseline plasma NfL and longitudinal changes in ADAS-Cog scores without significant impact on longitudinal hippocampal and WMH volumes (Table 2, see Supplementary Table 8 for detailed parameter estimates). Compared to non-DM group, DM group had more noticeable changes in the slopes of ADAS-Cog scores as plasma NfL level increased (Fig. 3C, left panel). Supplementary Table 9 displays the detailed parameters for slopes in Fig. 3C.

The association between baseline plasma NfL levels and longitudinal changes in ADAS-Cog scores, hippocampal volumes, and WMH volumes remained unaffected by impaired kidney function status (Table 2, see Supplementary Table 10 for detailed parameter estimates). The slopes of ADAS-Cog scores, hippocampal volumes, and WMH volumes increased with higher plasma NfL levels; however, these change rates did not differ significantly between participants with and without impaired kidney function (Fig. 3D and Supplementary Table 11).

The impact of obesity was significant only on the association between plasma NfL and longitudinal changes in hippocampal volumes; it did not significantly affect the associations with changes in ADAS-Cog scores or WMH volumes (Table 2, see Supplementary Table 12 for detailed parameter estimates). Obese participants presented more prominent changes in the slopes of hippocampal volumes compared to non-obese participants (middle panel of Fig. 3E, see Supplementary Table 13 for estimated monthly changes for outcome variables).

##### Sensitivity analysis: cardiometabolic risk factors as continuous variables

Supplementary Table 14 shows the results of linear mixed-effect models regarding the severity of cardiometabolic risk factors as continuous variables under the presence of each risk factor. Within the hypertension group, systolic blood pressure significantly moderated the association between plasma NfL and longitudinal WMH volume (beta -0.0014, *p*-value 0.004). Given that higher plasma NfL was associated with a faster increase in WMH volume (beta 0.002 in Supplementary Table 3), the negative beta value of -0.0014 indicates that as systolic blood pressure increased, the rate of increase in WMH volumes associated with higher plasma NfL levels was reduced. By contrast, systolic blood pressure did not significantly affect the association between plasma NfL and longitudinal ADAS-Cog score or hippocampal volume. Meanwhile, fasting glucose level in the DM group, eGFR in the impaired kidney function group, and BMI in the obesity group did not affect the association between plasma NfL and longitudinal changes in any outcomes.

## Discussion

In the ADNI cohort of 720 older adults without dementia, we observed a significant influence of Aβ on the association between baseline plasma NfL levels and changes in ADAS-Cog scores and hippocampal volumes. Among cardiometabolic risk factors (hypertension, DM, impaired kidney function, and obesity), the presence of hypertension had a significant impact on the capacity of plasma NfL for predicting longitudinal ADAS-Cog scores, hippocampal volumes, and WMH volumes. These findings suggest that the plasma NfL could be a valuable blood biomarker for predicting neurodegeneration and clinical progression in CU or MCI, particularly among older adults with Aβ or hypertension.

In the cross-sectional analysis, both lower eGFR and lower BMI were significantly associated with higher plasma NfL levels (Supplementary Table 2). However, cerebral Aβ, quantified as SUVR, systolic blood pressure, and fasting glucose level were not significantly associated with plasma NfL levels (Supplementary Table 2). These inverse cross-sectional associations between plasma NfL and eGFR or BMI are consistent with the previous studies [15, 19, 20, 39]. Although underlying mechanism remains unclear, it has been suggested that increased plasma NfL in individuals with lower eGFR may be due to reduced protein clearance [15, 19]. The inverse association between plasma NfL and BMI might be explained by either dilution from increased blood volume in individuals with higher BMI or by the neurodegenerative process, which can simultaneously provoke weight loss and NfL release [15, 39].

Longitudinally, higher plasma NfL levels predicted faster cognitive decline and changes in hippocampal and WMH volumes in non-demented participants (Fig. 2 and Supplementary Table 3). These findings, consistent with prior longitudinal studies [3–9], underscore the utility of plasma NfL as a blood biomarker for predicting clinical progression in older adults without dementia. Increased plasma NfL levels were associated with an accelerated rate of hippocampal volume loss, indicating that they can be an early sign of AD-specific neurodegeneration [3, 5–7]. Plasma NfL levels were also associated with longitudinal WMH volumes, aligning with the role of NfL as an early biomarker of cerebrovascular disease [18, 40]. Circulating NfL could be elevated due to subtle brain injury from subclinical cerebrovascular pathology [40]. Moreover, NfL reflects the damage to the axonal cytoskeleton, which comprises white matter integrity, leading to WMH [18, 40].

Of note, these prior longitudinal studies [3–9] did not consider the common old age-related conditions on the predictive performance of plasma NfL. Although some studies adjusted for renal function or history of major vascular events as covariates [4, 5], they did not evaluate whether these conditions altered the longitudinal association between plasma NfL and prospective neurodegeneration.

One of the novel aspects of our finding is the prognostic potential of plasma NfL for cognitive decline and hippocampal atrophy, particularly in the context of Aβ ( +) (Table 2, Supplementary Table 4, and Fig. 3A). A previous longitudinal study observed a more rapid increase in plasma NfL levels in the Aβ ( +) group compared to the Aβ ( −) group [14]. Our findings also align with a previous cross-sectional study that higher plasma NfL levels were associated with reduced grey matter density of AD-vulnerable regions only in individuals with Aβ ( +) [41]. Alongside prior findings, we suggest a possible interaction between Aβ and NfL; Aβ-induced early neuronal vulnerability may amplify the detrimental effects of axonal injury measured by NfL. A significant result in the hippocampus with a non-significant result in WMH suggests that this interaction between Aβ and NfL may be exerted in AD-related neurodegeneration rather than cerebral small vessel disease. Given that other pathologies can elevate NfL without cerebral Aβ deposition [2], participants with elevated NfL levels in company with Aβ may face a higher risk of neurodegeneration compared to those with elevated NfL alone. Baseline elevated plasma NfL in the absence of Aβ was possibly due to acute or temporary neuronal injury rather than progressive neurodegeneration, which might not have an association with longitudinal cognitive outcomes. However, a prior study observed that a temporary spiking with a subsequent decrease of blood NfL after acute brain injury still predicted longitudinal neurodegeneration [42]. Therefore, our results and this prior finding underscore the importance of considering Aβ status in the prognostic model based on plasma NfL, which is a useful blood-based biomarker for preventive clinical trials [12].

Compared to MCI participants, CU participants showed a more significant moderating effect of Aβ on the association between baseline plasma NfL and longitudinal ADAS-Cog score and WMH volume (Table 3). This finding suggests that the prognostic capacity of plasma NfL can be influenced by the status of Aβ, particularly in the earlier stage of AD. Previous cross-sectional studies showed that plasma NfL levels were comparable across amyloid status [3, 43], consistent with our result from the multiple regression model (Supplementary Table 2); however, plasma NfL levels were increased in MCI or dementia individuals compared to CU individuals [3, 43]. Therefore, the more obvious impact of Aβ in CU status suggests that amyloid pathology during the preclinical stage may enhance the prognostic capacity of plasma NfL by accelerating neurodegeneration, resulting in an increased circulating NfL during the prodromal stage. This speculation aligns with the pathophysiological process of AD, where amyloid deposition in CU status is followed by neurodegeneration, leading to cognitive decline in MCI status [44]. This is further supported by the observation that plasma NfL levels were higher in Aβ ( +) individuals than in Aβ ( −) individuals only in MCI status, not in CU status [3].

Among cardiovascular risk factors, hypertension appears to longitudinally amplify the potency of plasma NfL as a blood biomarker for neurodegeneration and clinical progression in older adults without dementia (Table 2, Supplementary Table 6, and Fig. 3B). Despite the strong association between hypertension and dementia-related neuroimaging biomarkers such as WMH [45, 46] or Aβ deposition [47], how hypertension would be related to plasma NfL is seldom investigated. A previous cross-sectional study observed no association between hypertension and plasma NfL level, consistent with our result (Supplementary Table 2) [15]. In our study, hypertension continued to influence the prognostic capacity of plasma NfL longitudinally. Hypertension exacerbates the cognitive decline and development of dementia in older adults [31]. Our result implies that hypertension-related cognitive decline can be explained by neurodegeneration or axonal injury expressed as plasma NfL. This interpretation is supported by a previous mouse study that hypertension accelerated cognitive decline, accompanied by AD pathologies, such as Aβ deposition and cerebral amyloid angiopathy, which led to hippocampal neurodegeneration [48]. A recent report from the Rotterdam study demonstrated that individuals with hypertension were associated with increased Aβ deposition after 7 years [49]. In addition to AD pathology, hypertension-related cardiovascular diseases induce microvascular injury in cerebral white matter [45, 46], which can increase NfL release via neuroaxonal damage [18, 40]. Our finding, together with these previous studies, implies the probable interaction between plasma NfL and hypertension.

Within the hypertension group, higher systolic blood pressure lessened the prognostic capacity of plasma NfL on WMH volume (Supplementary Table 14). This counterintuitive finding is in line with a previous intervention study that lowering blood pressure elevated plasma NfL in patients with hypertension, possibly due to reduced renal clearance [50]. In this study participants with hypertension, increased blood pressure might have introduced a decrease in plasma NfL, resulting in reduced NfL-related WMH change. Otherwise, in the state of higher systolic blood pressure, vascular or inflammatory pathologies, not reflected by NfL, may substantially contribute to increasing WMH volume. Meanwhile, systolic blood pressure did not influence the association between plasma NfL and longitudinal ADAS-Cog score and hippocampal volume (Supplementary Table 14). These non-significant findings suggest that the prognostic capacity of plasma NfL can be affected by hypertension-related cardiovascular conditions, such as myocardial infarction or atrial fibrillation [15], and not merely by systolic blood pressure alone. Moreover, as blood pressure was measured only once in this study, white-coat hypertension or transient hypotension due to blood pressure variability could not be excluded. Further longitudinal studies with comprehensive data on cardiovascular conditions, such as creatine kinase myocardial band, troponin-I, or electrocardiogram, will be helpful.

In contrast to Aβ and hypertension, DM affected the prognostic capacity of plasma NfL only in relation to changes in ADAS-Cog scores; however, it did not significantly impact hippocampal and WMH volumes (Table 2, Supplementary Table 8, and Fig. 3C). DM is associated with an accelerated cognitive decline and an increased risk of dementia [31, 51]. Our result of ADAS-Cog indicates that neurodegeneration or axonal injury, measured by NfL, can underpin DM-related cognitive decline. However, within the DM group, fasting glucose level was not associated with the prognostic capacity of plasma NfL (Supplementary Table 14). Given that hypoglycemia in DM also increases the risk of dementia [35], further investigations using other parameters reflecting DM conditions, such as hemoglobin A1c or glycemic variability, will be helpful. Meanwhile, our non-significant finding on hippocampal volume is consistent with a previous observation indicating no association between DM and AD pathology [52]. By contrast, it diverged from a recent finding from the Rotterdam Study, which indicated that DM predicted increased brain Aβ pathology after 7 years [49]. Our study tracked a 5-year trajectory of the hippocampus, not Aβ, potentially requiring a longer time to reveal the effect of diabetic status. Given the substantial inconstancy of the relationship between DM and AD pathology, further longitudinal studies with longer observation periods can be helpful. Albeit vascular pathology significantly contributes to dementia progression in DM [51], our finding implies that the prognostic model of plasma NfL for WMH does not need to consider diabetic status. This study did not measure other manifestations of small vessel diseases, such as lacunes, perivascular spaces, and cerebral microbleeds. Brain microinjury not captured by NfL, such as neuroinflammation, could ameliorate the effect of DM on predicting WMH volumes by baseline NfL. Moreover, our study focused on volumetric changes in the hippocampus and WMH, common features of AD-related dementia progression [3, 25], rather than examining changes in other brain regions. The impact of DM on plasma NfL may manifest in other brain regions. Furthermore, DM could provoke chronic injury in peripheral neurons and the central nervous system, which could affect plasma NfL levels [53]. Certain anti-diabetic medications, such as pioglitazone or metformin, can also delay cognitive decline or prevent dementia [54, 55]. These various conditions for DM participants, which were not considered in our study, might have introduced confounding factors.

 Impaired kidney function did not affect the prognostic capacity of plasma NfL for any of ADAS-Cog scores, hippocampal volumes, or WMH volumes (Table 2, Supplementary Table 10, and Fig. 3D). Furthermore, eGFR as a continuous variable also did not affect the prognostic capacity of plasma NfL within the impaired kidney function group (Supplementary Table 14). Despite observing that compromised kidney function can elevate plasma NfL levels (Supplementary Table 2) [15, 19, 56], our findings indicate that kidney function may not be a crucial consideration for the prognostic value of plasma NfL. A recent meta-analysis revealed that impaired kidney function is modestly associated with an increased risk of dementia [57]. However, our finding implies that this relationship may stem from underlying pathologies not significantly detectable by plasma NfL. Our result supports a previous study in which kidney function did not affect the correlation between plasma NfL levels and brain structure [58]. Otherwise, these non-significant results may be due to the characteristics of our study sample. For instance, we observed participants for approximately 5 years, which may not be sufficient to capture the impact of impaired kidney function. Moreover, individuals with medical conditions that could substantially affect cognition were excluded, potentially introducing selection bias. Additional longitudinal studies are required to overcome these shortcomings.

Obesity solely influenced the prognostic capacity of plasma NfL on hippocampal volumes without a significant impact on ADAS-Cog scores and WMH volumes (Table 2, Supplementary Table 12, and Fig. 3E). The result of hippocampus is consistent with a previous study that obesity is associated with pronounced hippocampal atrophy [59]. Furthermore, another study reported that increased BMI in midlife was associated with a faster increase in plasma NfL levels [60]. However, while midlife obesity is associated with an increased risk of dementia [31], late-life obesity does not have the same implications [37]. Rather, weight loss in older adults is associated with an increased risk of dementia [37]. This complexity might account for the non-significant results of ADAS-Cog scores and WMH volumes in our study participants, who had a mean age of 72.1. Besides, the significance of the result in hippocampal volume was lost when assessing the impact of BMI within obese participants (Supplementary Table 14). This can be explained by our previous finding that, although BMI loss was associated with the increased risk of dementia, obesity appeared to counteract this risk [38]. Otherwise, as participants needed to attend the clinic repetitively, those with severe obesity or cachexia may have been excluded or lost in follow-up, potentially contributing to non-significant findings. Additional large cohort studies are needed to elucidate the relationship between obesity, plasma NfL, neurodegeneration, and clinical progression.

Blood-based biomarkers for AD are valued for their non-invasiveness and cost-effectiveness compared to conventional AD biomarkers [1]. However, their application in real-world clinical practice is challenging owing to the influence of common conditions in older adults [1]. The results of this study indicate that a plasma NfL-based prognostic model for neurodegeneration and clinical progression needs to consider the status of Aβ and hypertension. For instance, an older adult with normal cognition but elevated plasma NfL level is at an increased risk of cognitive decline within a few years, particularly if Aβ or hypertension coexists. While DM and obesity may have an uncertain impact on the prognostic capacity of plasma NfL, impaired kidney function does not seem to affect this capacity.

To our knowledge, this is the first study to explore the impact of the potential moderating factors of AD dementia (Aβ and cardiometabolic risk factors) on the prognostic capacity of plasma NfL concerning neurodegeneration and clinical progression evaluated by cognitive function and neuroimagings. We investigated a relatively large prospective cohort observed over 5 years. Moreover, we evaluated the prognostic capacity of plasma NfL only among older adults without dementia, who are practical candidates for the application of blood-based biomarkers. Given that the study sample excluded individuals with severe medical conditions that could disrupt cognitive assessment, the impact of Aβ and hypertension on the prognostic capacity of plasma NfL may be more significant in real-world clinical practice.This study has limitations to consider when interpreting the results. First, excluding participants who did not have ADAS-Cog scores, MRI scans, or PET scans may have introduced selection bias. Second, this study used the data from a single ADNI cohort, which might limit the generalizability of our results despite the relatively large sample size. Our study sample predominantly consisted of White (*n* = 663, 92.1%) with a high level of education (mean 16.4 years). Considering the racial disparity in the prevalence of cardiometabolic conditions [61], replicative studies from diverse cohorts need to be performed. Moreover, ADNI excluded individuals with substantial cerebrovascular burden, which can affect plasma NfL levels [15]. This exclusion enhances the homogeneity of our study sample but also limits the application of the study results to individuals with a history of major cerebrovascular disease in real-world clinical practice. Third, the presence of hypertension, DM, and impaired kidney function was partially based on self-report, potentially limiting the accuracy of the diagnosis. The ADNI procedure manual instructed the investigators to review medical records submitted by participants. Both prescription and over-the-counter medications were also checked, and medical conditions necessitating these medications were recorded. Fourth, the precise assessment of hypertension and DM statuses was challenging. Blood pressure measurement, which was not taken repeatedly and only obtained while participants were sitting, could have led to inaccuracies. The severity of DM could not be determined due to the unavailability of hemoglobin A1c. A single measurement of fasting glucose may be insufficient to reflect diabetic status. Further studies with more thorough assessments are required. Lastly, given that the ADNI cohort could mainly consist of individuals with cognitive concerns, the study sample may not represent the real-world population.

## Conclusions

In conclusion, our study indicates that the prognostic capacity of plasma NfL for cognitive decline and dementia-related neuroimaging abnormalities is heightened when Aβ and hypertension coexist in our sample of non-demented older adults. Especially, the impact of Aβ was more prominent in CU participants than in MCI participants. The influence of DM and obesity on the predictive efficacy of plasma NfL appears less pronounced, whereas impaired kidney function may have a minimal effect. Consequently, when interpreting plasma NfL as a novel blood biomarker for the prognosis of progression of AD or other neurodegenerative diseases, it may be more informative to consider the coexistence of Aβ and hypertension.

## Supplementary Information

Supplementary Material 1.

## Data Availability

The data used in this study are from the ADNI database (http://adni.loni.usc.edu), which is accessible to interested scientists with the ADNI Data Use Agreement (http://adni.loni.usc.edu/wp-content/uploads/how_to_apply/ADNI_Data_Use_Agreement.pdf).

## Abbreviations

Aβ: Amyloid-β
AD: Alzheimer’s disease
ADAS-Cog: Alzheimer’s Disease Assessment Scale-Cognitive subscale
APOE: Apolipoprotein E
BMI: Body mass index
CU: Cognitively unimpaired
DM: Diabetes mellitus
eGFR: Estimated glomerular filtration rate
MCI: Mild cognitive impairment
MRI: Magnetic resonance imaging
NfL: Neurofilament light chain
PET: Positron emission tomography
SD: Standard deviation
SE: Standard error
SGDS: Short form of Geriatric Depression Scale
SUVR: Standard uptake value ratio
WMH: White matter hyperintensity
