# Supplementary material for: Impact of amyloid and cardiometabolic risk factors on prognostic capacity of plasma neurofilament light chain for neurodegeneration
Source: Alzheimers Res Ther. 2024 Sep 12;16:202. doi: 10.1186/s13195-024-01564-y (PMC11397040; doi:10.1186/s13195-024-01564-y)
Supplement: Supplementary file 1 — Supplementary Material 1. [file 13195_2024_1564_MOESM1_ESM.pdf]

Supplementary Tables

Title: Impact of amyloid and cardiometabolic risk factors on prognostic capacity of plasma neurofilament light chain for neurodegeneration

Table of Contents

Contents	Page
**Supplementary Table 1.** Number of participants who performed ADAS-Cog or MRI scan at each time point	1
**Supplementary Table 2.** Cross-sectional association of plasma NfL levels with Aβ and cardiometabolic risk factors	2
**Supplementary Table 3.** Association between baseline NfL levels and longitudinal changes in cognition/brain structure	3
**Supplementary Table 4**. Parameter estimates of linear mixed-effect models: Interaction between plasma NfL and Aβ	4
**Supplementary Table 5.** Estimated monthly change rates of cognition and brain structure across baseline plasma NfL levels: Stratified by Aβ status	5
**Supplementary Table 6.** Parameter estimates of linear mixed-effect models: Interaction between plasma NfL and hypertension	6
**Supplementary Table 7.** Estimated monthly change rates of cognition and brain structure across baseline plasma NfL levels: Stratified by hypertension status	7
**Supplementary Table 8.** Parameter estimates of linear mixed-effect models: Interaction between plasma NfL and DM	8
**Supplementary Table 9.** Estimated monthly change rates of cognition and brain structure across baseline plasma NfL levels: Stratified by DM status	9
**Supplementary Table 10.** Parameter estimates of linear mixed-effect models: Interaction between plasma NfL and impaired kidney function	10
**Supplementary Table 11.** Estimated monthly change rates of cognition and brain structure across baseline plasma NfL levels: Stratified by impaired kidney function status	11
**Supplementary Table 12.** Parameter estimates of linear mixed-effect models: Interaction between plasma NfL and obesity	12
**Supplementary Table 13.** Estimated monthly change rates of cognition and brain structure across baseline plasma NfL levels: Stratified by obesity status	13
**Supplementary Table 14.** Sensitivity analysis: impact of cardiometabolic risk factors as continuous variables under the presence of each risk factor	14

Supplementary Table 1. Number of participants who performed ADAS-Cog or MRI scan at each time point

Time	ADAS-Cog	MRI scan
Baseline	720	720
3 months		625
6 months	671	579
12 months	603	596
24 months	586	554
36 months	335	116
42 months	2	1
48 months	371	294
54 months	24	19
60 months	137	62
66 months	76	66
72 months	117	104
78 months	79	65
84 months	64	53
90 months	76	71
96 months	69	57
102 months	47	37
108 months	26	23
114 months	27	17
120 months	27	16
126 months	8	5
132 months	5	4

Abbreviations: ADAS-Cog, Alzheimer's disease assessment scale-cognitive subscale; MRI, magnetic resonance imaging.

Supplementary Table 2. Cross-sectional association of plasma NfL levels with Aβ and cardiometabolic risk factors

Explanatory variable	Outcome - NfL		
	beta	t-value	p-value
Florbetapir PET SUVR	0.018	0.516	0.606
Systolic blood pressure	-0.014	-0.453	0.651
Fasting glucose level	0.024	0.821	0.412
eGFR	-0.236	-6.850	<0.001
BMI	-0.152	-4.751	<0.001

Shown are results of a linear regression model where the outcome variable was plasma NfL level, and the main explanatory variables were Florbetapir PET SUVR, systolic blood pressure, fasting glucose level, eGFR, and BMI.
Continuous variables were standardized to z-scores.
The model was adjusted for baseline age, sex, years of education, APOE ε4 allele count, ever smoking, alcohol abuse, SGDS, ADAS-Cog score, hippocampal volume, WMH volume, and baseline cognitive status (MCI or CU).
Abbreviations: Aβ, amyloid-β; ADAS-Cog, Alzheimer's Disease Assessment Scale-Cognitive subscale; APOE, apolipoprotein E; BMI, body mass index; CU, cognitively unimpaired; DM, diabetes mellitus; eGFR, estimated glomerular filtration rate; NfL, neurofilament light chain; PET, positron emission tomography; SGDS, Short form of Geriatric Depression Scale; MCI, mild cognitive impairment; WMH, white matter hyperintensity.

Supplementary Table 3. Association between baseline NfL levels and longitudinal changes in cognition/brain structure

Explanatory variable	Outcome	beta	t-value	p-value
Plasma NfL × Time	ADAS-Cog	0.004	6.086	<0.001
	Hippocampal volume	-0.002	-5.713	<0.001
	WMH volume	0.002	4.584	<0.001

Shown are results of three linear mixed-effect models where the outcome variables were longitudinal ADAS-Cog score, hippocampal volume, and WMH volumes, respectively. In each model, the common main explanatory variable was the interaction term including plasma NfL and time. If this interaction term is statistically significant (p-value <0.05), the changes in outcomes over time is dependent on the plasma NfL levels.
Of the three outcome variables, ADAS-Cog score and WMH volume were square root transformed due to non-normal distribution.
Continuous variables except for time were standardized to z-scores.
Models were adjusted for following covariates: baseline age, sex, years of education, APOE ε4 allele count, ever smoking, alcohol abuse, SGDS, Aβ status, hypertension, DM, impaired kidney function, obesity, and baseline cognitive status (MCI or CU).
Abbreviations: ADAS-Cog, Alzheimer's disease assessment scale-cognitive subscale; APOE, apolipoprotein E; CU, cognitively unimpaired; DM, diabetes mellitus; MCI, mild cognitive impairment; NfL, neurofilament light chain; PET, positron emission tomography; SGDS, short form of Geriatric Depression Scale; SUVR, standard uptake value ratio; WMH, white matter hyperintensity.

Supplementary Table 4. Parameter estimates of linear mixed-effect models: Interaction between plasma NfL and Aβ

Explanatory variable	Outcome								
	ADAS-Cog			Hippocampal volume			WMH volume		
	beta	t-value	p-value	beta	t-value	p-value	beta	t-value	p-value
Intercept	-0.644	-7.702	<0.001	0.709	7.691	<0.001	-0.309	-3.467	<0.001
Plasma NfL	0.071	1.772	0.077	-0.041	-0.933	0.351	0.008	0.186	0.853
Time	0.002	2.391	0.017	-0.006	-18.496	<0.001	0.002	5.783	<0.001
Aβ	0.306	4.430	<0.001	-0.023	-0.303	0.762	0.262	3.546	<0.001
Age	0.205	5.659	<0.001	-0.242	-6.069	<0.001	0.334	8.669	<0.001
Sex	-0.335	-5.288	<0.001	-0.735	-10.537	<0.001	-0.090	-1.329	0.184
Education	-0.091	-2.920	0.004	-0.006	-0.164	0.870	-0.056	-1.686	0.092
APOE ε4 allele count	0.161	2.971	0.003	-0.186	-3.114	0.002	-0.017	-0.296	0.767
Smoking	-0.089	-1.130	0.259	0.059	0.679	0.497	0.007	0.084	0.933
Alcohol abuse	0.319	1.562	0.119	-0.311	-1.396	0.163	-0.358	-1.656	0.098
SGDS	0.056	1.737	0.083	-0.001	-0.015	0.988	0.004	0.107	0.915
Hypertension	0.006	0.089	0.929	-0.046	-0.640	0.522	0.201	2.867	0.004
DM	0.135	1.620	0.106	-0.134	-1.461	0.144	0.053	0.596	0.552
Impaired kidney function	0.038	0.275	0.784	-0.120	-0.784	0.433	-0.107	-0.721	0.471
Obesity	-0.001	-0.008	0.994	0.182	2.318	0.021	0.073	0.960	0.338
Baseline cognitive status (MCI or CU)	0.734	10.772	<0.001	-0.368	-4.903	<0.001	0.110	1.513	0.131
Plasma NfL × Time	0.002	2.783	0.006	-0.001	-2.724	0.007	0.001	2.679	0.008
Plasma NfL × Aβ	0.075	1.149	0.251	-0.048	-0.672	0.502	0.123	1.760	0.079
Time × Aβ	0.013	10.059	<0.001	-0.005	-9.572	<0.001	0.003	4.977	<0.001
**Plasma NfL × Time × Aβ**	0.004	2.797	0.005	-0.002	-2.922	0.004	0.001	1.408	0.160

Of the three outcome variables, ADAS-Cog score and WMH volume were square root transformed due to non-normal distribution.
Continuous variables except for time were standardized to z-scores.
Abbreviations: Aβ, amyloid-β; ADAS-Cog, Alzheimer's Disease Assessment Scale-Cognitive subscale; APOE, apolipoprotein E; CU, cognitively unimpaired; DM, diabetes mellitus; MCI, mild cognitive impairment; NfL, neurofilament light chain; SGDS, Short form of Geriatric Depression Scale; WMH, white matter hyperintensity.

Supplementary Table 5. Estimated monthly change rates of cognition and brain structure across baseline plasma NfL levels: Stratified by Aβ status

Aβ status	ADAS-Cog		Hippocampal volume		WMH volume	
	beta	SE	beta	SE	beta	SE
Aβ (+)						
Mean -1 SD NfL level	0.0087	0.0022	-0.0086	0.0008	0.0037	0.0009
Mean NfL level	0.0150	0.0013	-0.0111	0.0004	0.0057	0.0005
Mean +1 SD NfL level	0.0213	0.0019	-0.0135	0.0007	0.0076	0.0009
Aβ (−)						
Mean -1 SD NfL level	0.0002	0.0007	-0.0052	0.0003	0.0016	0.0005
Mean NfL level	0.0019	0.0006	-0.0061	0.0003	0.0026	0.0004
Mean +1 SD NfL level	0.0036	0.0009	-0.0069	0.0004	0.0036	0.0006

Of the three outcome variables, ADAS-Cog score and WMH volume were square root transformed due to non-normal distribution.

Plasma NfL level and outcome variables were standardized to z-scores.

Abbreviations: Aβ, amyloid-β; ADAS-Cog, Alzheimer's Disease Assessment Scale-Cognitive subscale; NfL, neurofilament light chain; SD, standard deviation; SE, standard error; WMH, white matter hyperintensity.

Supplementary Table 6. Parameter estimates of linear mixed-effect models: Interaction between plasma NfL and hypertension

Explanatory variable	Outcome								
	ADAS-Cog			Hippocampal volume			WMH volume		
	beta	t-value	p-value	beta	t-value	p-value	beta	t-value	p-value
Intercept	-0.629	-7.506	<0.001	0.702	7.613	<0.001	-0.297	-3.314	<0.001
Plasma NfL	0.087	1.932	0.054	-0.089	-1.805	0.072	0.040	0.817	0.414
Time	0.006	5.531	<0.001	-0.008	-17.970	<0.001	0.003	6.379	<0.001
Hypertension	0.004	0.067	0.946	-0.043	-0.597	0.550	0.208	2.935	0.003
Age	0.207	5.660	<0.001	-0.250	-6.227	<0.001	0.340	8.721	<0.001
Sex	-0.336	-5.286	<0.001	-0.734	-10.527	<0.001	-0.089	-1.312	0.190
Education	-0.088	-2.837	0.005	-0.005	-0.141	0.888	-0.054	-1.637	0.102
APOE ε4 allele count	0.169	3.111	0.002	-0.195	-3.274	0.001	-0.008	-0.139	0.889
Smoking	-0.094	-1.194	0.233	0.061	0.703	0.482	-0.001	-0.012	0.991
Alcohol abuse	0.327	1.602	0.110	-0.312	-1.397	0.163	-0.349	-1.612	0.107
SGDS	0.056	1.730	0.084	-0.001	-0.016	0.988	0.004	0.124	0.902
Aβ	0.282	4.072	<0.001	-0.008	-0.107	0.915	0.224	3.040	0.002
DM	0.148	1.769	0.077	-0.134	-1.462	0.144	0.064	0.720	0.472
Impaired kidney function	0.058	0.417	0.677	-0.144	-0.945	0.345	-0.070	-0.476	0.634
Obesity	-0.004	-0.057	0.954	0.190	2.421	0.016	0.068	0.892	0.373
Baseline cognitive status (MCI or CU)	0.739	10.810	<0.001	-0.377	-5.010	<0.001	0.118	1.623	0.105
Plasma NfL × Time	0.002	2.373	0.018	-0.001	-2.085	0.038	0.001	1.486	0.138
Plasma NfL × Hypertension	0.013	0.202	0.840	0.069	1.018	0.309	0.014	0.214	0.831
Time × Hypertension	0.002	1.648	0.100	0.000	-0.813	0.416	0.001	1.123	0.262
**Plasma NfL × Time × Hypertension**	0.005	3.606	<0.001	-0.002	-3.814	<0.001	0.002	3.389	<0.001

Of the three outcome variables, ADAS-Cog score and WMH volume were square root transformed due to non-normal distribution.
Continuous variables except for time were standardized to z-scores.
Abbreviations: Aβ, amyloid-β; ADAS-Cog, Alzheimer's Disease Assessment Scale-Cognitive subscale; APOE, apolipoprotein E; CU, cognitively unimpaired; DM, diabetes mellitus; MCI, mild cognitive impairment; NfL, neurofilament light chain; SGDS, Short form of Geriatric Depression Scale; WMH, white matter hyperintensity.

Supplementary Table 7. Estimated monthly change rates of cognition and brain structure across baseline plasma NfL levels: Stratified by hypertension status

Hypertension status	ADAS-Cog		Hippocampal volume		WMH volume	
	beta	SE	beta	SE	beta	SE
Hypertension (+)						
Mean -1 SD NfL level	0.0012	0.0012	-0.0057	0.0005	0.0013	0.0006
Mean NfL level	0.0084	0.0008	-0.0086	0.0003	0.0044	0.0004
Mean +1 SD NfL level	0.0157	0.0014	-0.0115	0.0006	0.0074	0.0007
Hypertension (−)						
Mean -1 SD NfL level	0.0041	0.0016	-0.0074	0.0006	0.0026	0.0007
Mean NfL level	0.0064	0.0013	-0.0081	0.0005	0.0033	0.0005
Mean +1 SD NfL level	0.0086	0.0017	-0.0089	0.0006	0.0039	0.0007

Of the three outcome variables, ADAS-Cog score and WMH volume were square root transformed due to non-normal distribution.
Plasma NfL level and outcome variables were standardized to z-scores.
Abbreviations: ADAS-Cog, Alzheimer's Disease Assessment Scale-Cognitive subscale; NfL, neurofilament light chain; SD, standard deviation; SE, standard error; WMH, white matter hyperintensity.

Supplementary Table 8. Parameter estimates of linear mixed-effect models: Interaction between plasma NfL and DM

Explanatory variable	Outcome								
	ADAS-Cog			Hippocampal volume			WMH volume		
	beta	t-value	p-value	beta	t-value	p-value	beta	t-value	p-value
Intercept	-0.628	-7.504	<0.001	0.702	8.355	<0.001	-0.290	-3.281	0.001
Plasma NfL	0.075	2.078	0.038	-0.060	-1.652	0.099	0.057	1.497	0.135
Time	0.007	9.943	<0.001	-0.008	-28.341	<0.001	0.004	10.909	<0.001
DM	0.145	1.744	0.082	-0.139	-1.662	0.097	0.066	0.744	0.457
Age	0.212	5.854	<0.001	-0.244	-6.726	<0.001	0.337	8.832	<0.001
Sex	-0.337	-5.323	<0.001	-0.733	-11.540	<0.001	-0.090	-1.348	0.178
Education	-0.089	-2.869	0.004	-0.007	-0.211	0.833	-0.054	-1.647	0.100
APOE ε4 allele count	0.170	3.142	0.002	-0.192	-3.543	<0.001	-0.008	-0.138	0.890
Smoking	-0.092	-1.181	0.238	0.062	0.785	0.433	-0.003	-0.038	0.970
Alcohol abuse	0.330	1.619	0.106	-0.317	-1.560	0.119	-0.345	-1.613	0.107
SGDS	0.057	1.756	0.080	0.000	-0.009	0.993	0.004	0.116	0.908
Aβ	0.271	3.907	<0.001	-0.009	-0.136	0.892	0.225	3.079	0.002
Hypertension	0.009	0.129	0.897	-0.045	-0.681	0.496	0.199	2.862	0.004
Impaired kidney function	0.067	0.483	0.629	-0.130	-0.941	0.347	-0.079	-0.544	0.586
Obesity	-0.003	-0.037	0.970	0.184	2.575	0.010	0.069	0.919	0.358
Baseline cognitive status (MCI or CU)	0.743	10.892	<0.001	-0.373	-5.455	<0.001	0.118	1.637	0.102
Plasma NfL × Time	0.004	4.775	<0.001	-0.001	-5.041	<0.001	0.001	4.127	<0.001
Plasma NfL × DM	0.158	1.695	0.090	0.015	0.164	0.870	-0.055	-0.561	0.575
Time × DM	0.003	1.537	0.125	0.000	0.035	0.972	0.000	0.296	0.767
**Plasma NfL × Time × DM**	0.008	3.436	<0.001	-0.001	-1.296	0.195	0.001	0.861	0.390

Of the three outcome variables, ADAS-Cog score and WMH volume were square root transformed due to non-normal distribution.
Continuous variables except for time were standardized to z-scores.
Abbreviations: Aβ, amyloid-β; ADAS-Cog, Alzheimer's Disease Assessment Scale-Cognitive subscale; APOE, apolipoprotein E; CU, cognitively unimpaired; DM, diabetes mellitus; MCI, mild cognitive impairment; NfL, neurofilament light chain; SGDS, Short form of Geriatric Depression Scale; WMH, white matter hyperintensity.

Supplementary Table 9. Estimated monthly change rates of cognition and brain structure across baseline plasma NfL levels: Stratified by DM status

DM status	ADAS-Cog		Hippocampal volume		WMH volume	
	beta	SE	beta	SE	beta	SE
DM (+)						
Mean -1 SD NfL level	-0.0021	0.0029	-0.0057	0.0009	0.0023	0.0013
Mean NfL level	0.0101	0.0019	-0.0083	0.0006	0.0044	0.0009
Mean +1 SD NfL level	0.0224	0.0034	-0.0109	0.0011	0.0065	0.0016
DM (−)						
Mean -1 SD NfL level	0.0037	0.0010	-0.0069	0.0004	0.0022	0.0005
Mean NfL level	0.0073	0.0007	-0.0084	0.0003	0.0038	0.0003
Mean +1 SD NfL level	0.0109	0.0011	-0.0099	0.0004	0.0054	0.0005

Of the three outcome variables, ADAS-Cog score and WMH volume were square root transformed due to non-normal distribution.
Plasma NfL level and outcome variables were standardized to z-scores.
Abbreviations: ADAS-Cog, Alzheimer's Disease Assessment Scale-Cognitive subscale; NfL, neurofilament light chain; SD, standard deviation; SE, standard error; WMH, white matter hyperintensity.

Supplementary Table 10. Parameter estimates of linear mixed-effect models: Interaction between plasma NfL and impaired kidney function

Explanatory variable	Outcome								
	ADAS-Cog			Hippocampal volume			WMH volume		
	beta	t-value	p-value	beta	t-value	p-value	beta	t-value	p-value
Intercept	-0.622	-7.457	<0.001	0.704	7.642	<0.001	-0.286	-3.205	0.001
Plasma NfL	0.109	3.047	0.002	-0.046	-1.174	0.241	0.058	1.527	0.127
Time	0.008	11.002	<0.001	-0.008	-29.954	<0.001	0.004	11.154	<0.001
Impaired kidney function	0.156	0.946	0.344	-0.012	-0.068	0.945	0.043	0.242	0.809
Age	0.209	5.788	<0.001	-0.242	-6.090	<0.001	0.338	8.774	<0.001
Sex	-0.339	-5.365	<0.001	-0.738	-10.591	<0.001	-0.094	-1.385	0.167
Education	-0.091	-2.924	0.004	-0.009	-0.262	0.793	-0.056	-1.682	0.093
APOE ε4 allele count	0.174	3.211	0.001	-0.187	-3.142	0.002	-0.006	-0.104	0.917
Smoking	-0.095	-1.212	0.226	0.062	0.726	0.468	0.000	-0.003	0.998
Alcohol abuse	0.327	1.605	0.109	-0.321	-1.444	0.149	-0.350	-1.619	0.106
SGDS	0.057	1.779	0.076	0.001	0.014	0.989	0.005	0.155	0.877
Aβ	0.277	4.012	<0.001	-0.009	-0.116	0.908	0.216	2.933	0.003
Hypertension	-0.001	-0.010	0.992	-0.050	-0.691	0.490	0.200	2.841	0.005
DM	0.141	1.696	0.090	-0.148	-1.618	0.106	0.058	0.656	0.512
Obesity	-0.004	-0.050	0.960	0.185	2.369	0.018	0.072	0.955	0.340
Baseline cognitive status (MCI or CU)	0.743	10.924	<0.001	-0.369	-4.922	<0.001	0.118	1.623	0.105
Plasma NfL × Time	0.005	6.034	<0.001	-0.002	-5.393	<0.001	0.001	3.784	<0.001
Plasma NfL × Impaired kidney function	-0.107	-1.055	0.292	-0.127	-1.141	0.254	-0.100	-0.915	0.361
Time × Impaired kidney function	0.000	-0.008	0.994	0.001	0.769	0.443	0.002	0.930	0.353
**Plasma NfL × Time × Impaired kidney function**	-0.002	-0.597	0.551	-0.001	-0.480	0.632	0.002	1.502	0.133

Of the three outcome variables, ADAS-Cog score and WMH volume were square root transformed due to non-normal distribution.
Continuous variables except for time were standardized to z-scores.
Abbreviations: Aβ, amyloid-β; ADAS-Cog, Alzheimer's Disease Assessment Scale-Cognitive subscale; APOE, apolipoprotein E; CU, cognitively unimpaired; DM, diabetes mellitus; MCI, mild cognitive impairment; NfL, neurofilament light chain; SGDS, Short form of Geriatric Depression Scale; WMH, white matter hyperintensity.

Supplementary Table 11. Estimated monthly change rates of cognition and brain structure across baseline plasma NfL levels: Stratified by impaired kidney function status

Impaired kidney function status	ADAS-Cog		Hippocampal volume		WMH volume	
	beta	SE	beta	SE	beta	SE
Impaired kidney function (+)						
Mean -1 SD NfL level	0.0035	0.0032	-0.0051	0.0022	0.0019	0.0035
Mean NfL level	0.0064	0.0019	-0.0073	0.0014	0.0047	0.0020
Mean +1 SD NfL level	0.0093	0.0020	-0.0095	0.0013	0.0076	0.0025
Impaired kidney function (−)						
Mean -1 SD NfL level	0.0031	0.0010	-0.0068	0.0004	0.0024	0.0004
Mean NfL level	0.0077	0.0007	-0.0084	0.0003	0.0039	0.0003
Mean +1 SD NfL level	0.0122	0.0011	-0.0100	0.0004	0.0054	0.0005

Of the three outcome variables, ADAS-Cog score and WMH volume were square root transformed due to non-normal distribution.
Plasma NfL level and outcome variables were standardized to z-scores.
Abbreviations: ADAS-Cog, Alzheimer's Disease Assessment Scale-Cognitive subscale; NfL, neurofilament light chain; SD, standard deviation; SE, standard error; WMH, white matter hyperintensity.

Supplementary Table 12. Parameter estimates of linear mixed-effect models: Interaction between plasma NfL and obesity

Explanatory variable	Outcome								
	ADAS-Cog			Hippocampal volume			WMH volume		
	beta	t-value	p-value	beta	t-value	p-value	beta	t-value	p-value
Intercept	-0.620	-7.414	<0.001	0.704	7.630	<0.001	-0.277	-3.110	0.002
Plasma NfL	0.087	2.427	0.015	-0.074	-1.895	0.058	0.024	0.634	0.526
Time	0.008	10.142	<0.001	-0.009	-27.506	<0.001	0.004	10.819	<0.001
Obesity	0.007	0.091	0.927	0.217	2.693	0.007	0.097	1.235	0.217
Age	0.204	5.624	<0.001	-0.249	-6.233	<0.001	0.330	8.536	<0.001
Sex	-0.338	-5.337	<0.001	-0.736	-10.564	<0.001	-0.093	-1.380	0.168
Education	-0.089	-2.871	0.004	-0.007	-0.198	0.843	-0.055	-1.664	0.097
APOE ε4 allele count	0.170	3.152	0.002	-0.188	-3.167	0.002	-0.008	-0.131	0.896
Smoking	-0.093	-1.195	0.232	0.065	0.756	0.450	0.000	0.004	0.997
Alcohol abuse	0.332	1.630	0.103	-0.315	-1.413	0.158	-0.342	-1.586	0.113
SGDS	0.058	1.800	0.072	0.003	0.082	0.935	0.009	0.251	0.802
Aβ	0.277	4.015	<0.001	-0.007	-0.095	0.924	0.216	2.948	0.003
Hypertension	0.006	0.089	0.929	-0.041	-0.562	0.574	0.210	2.996	0.003
DM	0.147	1.772	0.077	-0.143	-1.557	0.120	0.065	0.731	0.465
Impaired kidney function	0.049	0.354	0.723	-0.153	-1.002	0.317	-0.099	-0.670	0.503
Baseline cognitive status (MCI or CU)	0.735	10.755	<0.001	-0.381	-5.059	<0.001	0.106	1.456	0.146
Plasma NfL × Time	0.004	4.875	<0.001	-0.001	-4.193	<0.001	0.001	3.354	<0.001
Plasma NfL × Obesity	0.083	0.947	0.344	0.134	1.399	0.162	0.203	2.185	0.029
Time × Obesity	-0.001	-0.355	0.723	0.001	1.128	0.260	0.000	-0.452	0.652
**Plasma NfL × Time × Obesity**	0.003	1.594	0.112	-0.002	-2.238	0.026	0.002	1.898	0.058

Of the three outcome variables, ADAS-Cog score and WMH volume were square root transformed due to non-normal distribution.
Continuous variables except for time were standardized to z-scores.
Abbreviations: Aβ, amyloid-β; ADAS-Cog, Alzheimer's Disease Assessment Scale-Cognitive subscale; APOE, apolipoprotein E; CU, cognitively unimpaired; DM, diabetes mellitus; MCI, mild cognitive impairment; NfL, neurofilament light chain; SGDS, Short form of Geriatric Depression Scale; WMH, white matter hyperintensity.

Supplementary Table 13. Estimated monthly change rates of cognition and brain structure across baseline plasma NfL levels: Stratified by obesity status

Obesity status	ADAS-Cog		Hippocampal volume		WMH volume	
	beta	SE	beta	SE	beta	SE
Obesity (+)						
Mean -1 SD NfL level	0.0006	0.0012	-0.0047	0.0006	0.0007	0.0007
Mean NfL level	0.0065	0.0009	-0.0079	0.0005	0.0036	0.0005
Mean +1 SD NfL level	0.0125	0.0019	-0.0110	0.0010	0.0065	0.0011
Obesity (−)						
Mean -1 SD NfL level	0.0042	0.0013	-0.0075	0.0005	0.0026	0.0005
Mean NfL level	0.0081	0.0009	-0.0087	0.0003	0.0040	0.0004
Mean +1 SD NfL level	0.0121	0.0012	-0.0100	0.0005	0.0054	0.0005

Of the three outcome variables, ADAS-Cog score and WMH volume were square root transformed due to non-normal distribution.
Plasma NfL level and outcome variables were standardized to z-scores.
Abbreviations: ADAS-Cog, Alzheimer's Disease Assessment Scale-Cognitive subscale; NfL, neurofilament light chain; SD, standard deviation; SE, standard error; WMH, white matter hyperintensity.

Supplementary Table 14. Sensitivity analysis: impact of cardiometabolic risk factors as continuous variables under the presence of each risk factor

Explanatory variable	Outcome	beta	t value	p-value
Plasma NfL × Time × Systolic blood pressure (Within hypertension group)	ADAS-Cog score	-0.0004	-0.412	0.681
	Hippocampal volume	0.0006	1.426	0.155
	WMH volume	-0.0014	-2.874	0.004
Plasma NfL × Time × Fasting glucose level (Within DM group)	ADAS-Cog score	0.0014	0.968	0.336
	Hippocampal volume	-0.0001	-0.177	0.860
	WMH volume	-0.0002	-0.244	0.807
Plasma NfL × Time × eGFR (Within impaired kidney function group)	ADAS-Cog score	0.0021	0.570	0.572
	Hippocampal volume	-0.0010	-0.637	0.526
	WMH volume	-0.0009	-0.260	0.796
Plasma NfL × Time × BMI (Within obesity group)	ADAS-Cog score	0.0002	0.173	0.863
	Hippocampal volume	0.0001	0.194	0.847
	WMH volume	0.0004	0.464	0.644

Shown are results of linear mixed-effect models where each main explanatory variable was the three-way interaction term including baseline NfL, time, and the variable of interest (systolic blood pressure, fasting glucose level, eGFR, or BMI). If the interaction term is statistically significant (p-value <0.05), the association between plasma NfL and longitudinal changes in outcome is dependent on the status of the variable of interest (systolic blood pressure, fasting glucose level, eGFR, or BMI).
Of the three outcome variables, ADAS-Cog score and WMH volume were square root transformed due to non-normal distribution.
Continuous variables except for time were standardized to z-scores.
All models were adjusted for the following covariates: baseline age, sex, years of education, APOE ε4 allele count, ever smoking, alcohol abuse, SGDS, Aβ status, systolic blood pressure, fasting glucose level, BMI, eGFR, and baseline cognitive status (MCI or CU).

Abbreviations: Aβ, amyloid-β; ADAS-Cog, Alzheimer's Disease Assessment Scale-Cognitive subscale; APOE, apolipoprotein E; BMI, body mass index; CU, cognitively unimpaired; DM, diabetes mellitus; eGFR, estimated glomerular filtration rate; MCI, mild cognitive impairment; NfL, neurofilament light chain; SGDS, Short form of Geriatric Depression Scale; WMH, white matter hyperintensity.
